# Antithrombotic therapy at the end-of-life—continue or stop?

**DOI:** 10.1016/j.rpth.2026.103459

**Published:** 2026-03-26

**Authors:** Gerard Gurumurthy, Naisthika Kumar, Lianna Reynolds, Biddy Bassam, Marc Carrier, Simon Noble, Alok A. Khorana, Jecko Thachil

**Affiliations:** 1 The Queen Elizabeth Hospital Kings Lynn NHS Foundation Trust, King’s Lynn, UK; 2 Addenbrooke’s Hospital, Cambridge University Hospitals NHS Foundation Trust, Cambridge, UK; 3 Department of Haematology, Manchester University NHS Foundation Trust, Manchester, UK; 4 Department of Palliative Care, The Queen Elizabeth Hospital Kings Lynn NHS Foundation Trust, King’s Lynn, UK; 5 Department of Medicine, University of Ottawa, The Ottawa Hospital Research Institute, Ottawa, Ontario, Canada; 6 Marie Curie Palliative Care Research Centre, Cardiff University, Cardiff, UK; 7 Cleveland Clinic Taussig Cancer Center, Department of Hematology Oncology, Ohio, USA; 8 Case Comprehensive Cancer Center, Cleveland, Ohio, USA; 9 Manchester Academic Health Science Centre (MAHSC), The University of Manchester, Manchester, UK

**Keywords:** anticoagulation, antithrombotic, end-of-life, palliative, venous thromboembolism

## Abstract

Antithrombotics at the end-of-life pose a clinical challenge where indication-specific time-to-benefit, bleeding risk, and patient priorities must be reconciled over short prognostic horizons. In this review of the literature, we found that a substantial number of individuals remain on anticoagulants and antiplatelet therapy till the point of death. Prospective ultrasound surveillance studies show a high baseline incidence of asymptomatic proximal deep vein thrombosis at admission but a low short-term incidence of new events during typical hospice stays. Additionally, large home palliative cohorts suggest that deprescribing anticoagulants does not increase clinical thrombosis and may reduce bleeding and facilitate home death. Bleeding on antithrombotics is also common in the last months of life. Clinically relevant bleeding occurs in up to 1 in 10, and fatal hemorrhage has been reported. Cancer-specific factors, chronic or end-stage kidney disease, cytopenias, and drug interactions heighten the risks. Where anticoagulation is pursued, the choice of agent and route should reflect swallowing, nutrition, renal or hepatic function, monitoring capacity, and reversal intent. Structured, shared decision making and proactive deprescribing when benefits no longer outweigh harms are central.

## Essentials

• Antithrombotics are often continued near death despite uncertain short-term benefit.
• Deprescribing anticoagulants may reduce bleeding without increasing thrombosis.
• If pursued, anticoagulation requires regular review and a shared deprescribing plan.
• Shared decision making supports safe and goal-concordant deprescribing.

## 1 Introduction

Anticoagulants and antiplatelet agents often form part of the medication list in patients approaching the end-of-life. The decision when to initiate, continue, or discontinue this treatment can be a complex one. Venous thromboembolism (VTE) represents a major preventable cause of morbidity and mortality. Early and appropriate anticoagulation is the mainstay of treatment, but careful consideration needs to be given if there is a bleeding risk that formal anticoagulation could exacerbate. VTE contributes to substantial complications, including death and postembolic syndromes following deep vein thrombosis (DVT) [1]. However, antithrombotic therapy at the end-of-life care encompasses heterogeneous indications beyond VTE. This includes stroke prevention in atrial fibrillation (AF), thromboprophylaxis for mechanical heart valves, and antiplatelet therapy among many other indications, each of which has its own distinct time-to-benefit profiles and risk–benefit trade-offs as prognosis shortens. As such, there is an increased understanding of prevention in patients who may be at high risk of thrombosis as assessed using risk–benefit calculations and relevant risk assessment models [2].

For patients in the final stages of life, the decision to (dis)continue or initiate anticoagulation becomes more nuanced as these models were not derived from a palliative patient cohort, and often, a more pragmatic approach, involving the patient in the decision making, is required. Most randomized trials and many guidelines for anticoagulation have historically excluded patients with limited life expectancy or those receiving hospice or palliative care, which makes extrapolation to this distinct population uncertain and contributies to considerable practice variation. Furthermore, such studies used clinical outcome measures, which are of less relevance to patients nearing the end-of-life where symptom burden and quality of life takes primacy. Clinicians must balance the risk of VTE against the risk of bleeding on the background of patient’s preferences and priorities, often in an emotive context, and, if deemed essential to continue, consider the means of delivery such as subcutaneous injections when swallowing medication is no longer possible.

While deprescribing is not an emotionally neutral action, some patients and their families can perceive it as giving up as it demonstrates the shift in emphasis from quantity to quality of life [3]. Continuing unnecessary or burdensome treatment is also not an ethically neutral action. Treatment is often initiated within the ethical framework of beneficence and nonmaleficence. Yet, when quality of life becomes the priority, this can change how those principles are applied. Time-to-benefit and time-to-harm are particularly important in this context but can be difficult to weigh when prognosis is uncertain. Patient autonomy is also a crucial consideration although this too may be affected by impaired decision-making capacity. These ethical principles can also conflict: for example, when a patient wishes to continue a medication that is no longer beneficial and is causing harm. In the National Health Service, UK, this further raises the ethical question of justice, which addresses the principle that health care is a valuable resource to be distributed equitably. In the absence of clear protocols to nuance this risk, practice varies widely, and clinicians are unguided in these challenging decisions.

In this review, we define palliative care in line with the World Health Organization’s guidance to denote care for people with life-limiting illness in whom the focus is on quality of life and symptom control. We define end-of-life care for patients in the last months to year of life and last days of life for those who are actively dying. This distinction matters because the balance of thrombotic benefit, bleeding risk, and treatment burden changes across these phases.

## 2 Anticoagulation in the End-of-life Setting

Current UK National Institute for Care Excellence (NICE) guidelines advise to avoid initiating pharmacologic VTE prophylaxis in the last days of life and to review prophylaxis daily in palliative care, with explicit attention to patient and family views [4]. In addition, guidelines from the American Society of Clinical Oncology (ASCO) state that initiating anticoagulation is of uncertain benefit in patients receiving end-of-life or hospice care or with very limited life expectancy with no palliative or symptom reduction [5]. While these suggestions are helpful with respect to initiation of primary thromboprophylaxis in patients who are not receiving antithrombotics, there is a paucity of guidance for continuation, de-escalation, or cessation of therapeutic anticoagulation in patients already established on full-dose treatment nearing the end-of-life.

Consequently, a substantial population of individuals will remain anticoagulated in the final days of life (Table 1) [[6], [7], [8], [9], [10], [11]]. In a cross-sectional study of 15,217 US nursing-home residents with AF and advanced dementia, 33.1% received an anticoagulant during the last 6 months of life [6]. In multivariable modeling, greater use was associated with higher CHA_2_DS_2_-VASc of >7 (odds ratio [OR], 1.38; 95% CI, 1.23-1.54), higher anticoagulation and risk factors in atrial fibrillation (ATRIA) score of >7 (OR, 1.25; 95% CI, 1.13-1.39), >1 year nursing-home stay (OR, 2.68; 95% CI, 2.48-2.89), weight loss (OR, 1.09; 95% CI, 1.01-1.18), and difficulty in swallowing (OR, 1.12; 95% CI, 1.02-1.22). Lower use was associated with older age (80-89 years: OR, 0.82; 95% CI, 0.74-0.92; ≥90 years: OR, 0.59; 95% CI, 0.52-0.66), female sex (OR, 0.88; 95% CI, 0.81-0.95), and hospice enrolment (OR, 0.76; 95% CI, 0.70-0.83). Another study determined that patients who are actively treated for VTE or with a history of AF or aortic/mitral valve replacement were significantly more likely to receive antithrombotic therapy [8]. In a retrospective chart review (*n* = 180) to quantify real-world antithrombotic use in patients from primary care and institutional settings, it was found that 60% used an antithrombotic in the last 3 months of life, with 76% of users continuing until the final week [7]. One similar study found that a substantial proportion of patients continued antithrombotic therapy until death [11].

**Table 1:** Incidence of antithrombotic prescriptions.

Study	Sample size	Antithrombotic type	Setting or disease	Incidence of prescription
Ouellet et al. [6]	15,217	Anticoagulants for atrial fibrillation	Nursing home, advanced dementia, last 6 mo of life	33.1% received anticoagulant in last 6 mo of life
Huisman et al. [7]	180	Antithrombotics	Life-limiting illness (malignant or nonmalignant) in last 3 mo of life	60% used antithrombotics in last 3 mo; 75.9% of those continued until last week before death
Kowalewska et al. [8]	1141	Antithrombotics	Hospice discharge setting	6.7% had antithrombotic prescription at discharge to hospice care
Aldridge et al. [9]	25,783	Antithrombotics	Advanced cancer, end-of-life setting (median survival, 145 d)	32% receiving antithrombotics at diagnosis; 77% of those continued until death
Kempers et al. [10]	18,145	Vitamin K antagonists	Predominantly heart disease, hip fracture, and cancer	Cumulative incidence of discontinuation at 1 y was 14.0%, and >80% continued anticoagulants until the last month of life (median, 14 d between discontinuation and death)
Søgaard et al. [11]	86,732	Antithrombotics	Cancer	37.5% were receiving antithrombotic therapy at terminal illness declaration; most continued until death (74.8% of platelet inhibitor users, 58.8% of DOAC users, and 61.6% of VKA users)

AF, atrial fibrillation; DOAC, direct oral anticoagulant; VKA, vitamin K antagonist.

In addition, a retrospective Welsh cohort study (*n* = 25,783) found that 32% of patients were receiving antithrombotic therapy at the point of a poor cancer prognosis, with 77% continuing until the time of death [9]. The median survival in that cohort was 145 days. Lastly, a Dutch cohort study of 18,145 vitamin K antagonists’ users (median age, 81 years) with life-limiting disease (mainly heart disease, hip fracture and cancer) found that only 14.0% had anticoagulants discontinued at 1 year, with >80% of patients continuing treatment until the last month of life (median, 14 days before death) [10]. Other studies report a low continuation rate of anticoagulation. One retrospective chart review (*n* = 1141) found that only 77 individuals (6.7%) received a prescription for antithrombotic therapy on discharge to hospice care [8]. The rationale for continuing anticoagulation into the end-of-life setting is not always well documented. The same cohort study found that 54.5% did not have any documented rationale for continuation of anticoagulation upon discharge to hospice [8].

## 3 Why do Clinicians Continue Anticoagulation in the Palliative-care Setting?

### 3.1 Cancer-associated thrombosis scenario

There is a high rate of continuation of secondary thromboprophylaxis despite a degree of uncertainty of benefits, as mentioned in NICE and ASCO guidelines. There are several disease-specific associations that may compel clinicians to continue therapy in this population (Table 2). Patients with cancer, estimated to be around one-third of the palliative-care population [12], are at a 4- to 7-fold increased risk of developing a VTE [[13], [14], [15]]. The risk of cancer-associated venous thrombosis (CAT) is multifactorial; some cancers, such as pancreatic malignancies, are inherently thrombogenic, and chemoradiotherapy is associated with additional VTE risks [[16], [17], [18]]. Patients with cancers are thought to be highly thrombogenic at the end-of-life due to a combination of disease progression, immobility, dehydration, and infection [19]. There is also a strong risk of VTE recurrence even while on anticoagulation [20]. In the subgroup analysis of the Hokusai-VTE Cancer trial (edoxaban vs dalteparin), recurrent VTE occurred at ∼7.9% to 11.3% at 12 months, varying by tumor site and regimen [21]. In the CARAVAGGIO trial (apixaban vs dalteparin), recurrent VTE occurred at 5.6% vs 7.9% [22]. The 12-month extensions or pooled updates kept the 1-year recurrence ∼8% to 12% [22]. The overall rates of recurrent VTE varied from 1% to 12%, with the highest risk observed in the patient subgroup with residual vein thrombosis present at 6 months randomized to receive no anticoagulation (13%-15%) [23]. In a meta-analysis, the pooled rate of recurrent CAT was 14.6 per 100 person-years in the first 3 months from anticoagulation discontinuation [24]. Hence, clinicians may feel inclined to continue anticoagulation given the high recurrence rate in this population.

**Table 2:** Key drivers of continuation of antithrombotics at end-of-life.

Driver of continuation	Supporting evidence or examples	Underlying rationale
High perceived thrombotic risk (especially cancer-associated thrombosis)	Hokusai-VTE Cancer, CARAVAGGIO, and meta-analyses showing recurrence 8%-12% at 12 mo and 14.6 per 100 person-years after discontinuation	Clinicians may aim to prevent recurrent VTE and associated morbidity and mortality
Absence of clear discontinuation guidelines	Survey of mechanical valve anticoagulation, showing 94% desired guidance, and a UK clinician survey, showing 83% increased confidence if national guidance existed	Default continuation in absence of structured deprescribing pathways
Overestimation of short-term thrombosis risk	Survey data showing clinicians overestimated daily thrombosis risk after warfarin cessation in mechanical valves	Risk aversion and uncertainty favor continuation
Medicolegal concerns	National clinician survey reporting medicolegal worries influencing anticoagulation decisions	Fear of responsibility for preventable thrombotic events
Ethical and psychological drivers	Qualitative studies demonstrating decisions shaped by clinician moral frameworks and perceptions of acceptable death	Stopping therapy may be perceived as withdrawal of active care or giving up
Prognostic uncertainty	Realist synthesis demonstrating prescribing inertia due to uncertainty in prognosis and treatment benefit	Difficulty estimating life expectancy and time-to-benefit profiles
Fragmented ownership across care transitions	Observational chart reviews and transition-of-care studies demonstrating unclear prescribing responsibility	Continuation occurs when responsibility for reassessment is not well defined

VTE, venous thromboembolism.

It is crucial to emphasize that these recurrence estimates largely reflect longer time horizons (months to a year), whereas many patients in hospice or the last weeks to months of life have a short prognostic horizon. In this context, quality of life, symptom control, and treatment burden become the main considerations. In this study, the most clinically important question is not recurrence risk over 6 to 12 months but whether anticoagulation is likely to prevent a symptomatic event or meaningfully reduce symptom burden within the patient’s remaining lifetime and whether this benefit outweighs bleeding risk and the practical burdens of treatment. There is a paucity of data focusing on these considerations and may be quite individual to the patient.

### 3.2 Absence of specific guidelines

A lack of guidelines to support discontinuation of anticoagulation at this stage is also a contributing factor. In the above-mentioned survey of mechanical valve anticoagulation, 94% of respondents wanted guidance on the conundrum. As a result, clinicians may often seek advice from colleagues around anticoagulation at the end-of-life [25]. One survey of 186 UK clinicians found that up to 56% would do so. In that study, 83% of respondents stated they would feel more confident if national guidance existed [26]. In general, there is a paucity of guidelines regarding anticoagulant cessation, especially in this patient group. Short-term thrombotic risk assessment may often not be done, and this may contribute to continuation of therapy. This is seen in a UK survey on management of patients with mechanical valve nearing end-of-life, where 90% of respondents felt poorly informed and most overestimated short-term thrombosis risk if warfarin were stopped [25]. The same study reproduced risk tables showing much lower day-to-day risks than clinicians assumed.

### 3.3 Worries about medicolegal implications

In the absence of clear algorithmic approaches to guide anticoagulation in this group, clinicians report worrying about the medicolegal implications of discontinuing anticoagulation. Up to 50% may report that these worries influence their decision making [26]. In a qualitative study of UK doctors managing patients with CAT at the end-of-life, it was found that VTE treatment decisions were driven by individual physicians’ moral and ethical opinions [27]. Some clinicians considered treatment overcautious from themselves or others, again suggesting a more character based than evidence-based approach. There are some suggestions that decisions are being made on the basis of clinicians’ divergent opinions as to the virtues of different modes of death. Overall ambiguity appeared to abound, and it appears clear that without clear guidelines, decision to anticoagulate is compounded by ethical conundrums.

### 3.4 Prognostic uncertainty

Lastly, a recent realist synthesis of 91 studies explains the persistence of antithrombotic prescribing near the end-of-life through multilevel prescribing inertia that favors continuation over deprescribing [28]. The data set showed that clinicians face genuine prognostic uncertainty with limited evidence base for antithrombotic therapy at the end-of-life, which compound to make stopping feel riskier than continuing. The synthesis also found that clinicians often worry that raising deprescribing could be perceived by patients and families as giving up, although many patients are often pleased to have a discussion about their medication and reducing the tablet burden. Interprofessional dynamics, including deference to the original prescriber and the absence of consensus processes, and organizational barriers, including time pressure, unclear ownership, and electronic records that do not prompt goals-of-care or medication review, were found to reinforce the status quo. The authors conclude that in the absence of targeted supports such as shared decision-making tools and structured team pathways, antithrombotic therapy is usually continued into the last phase of life despite heightened bleeding risk and uncertain patient-centered benefit.

Prognostic challenges may also differ between disease groups [29]. In advanced cancer, several tools provide moderately accurate prognostic estimates [30]. It may be useful to signpost that prognosis estimation can be supported by validated tools in advanced cancer [31], which, while with its own limitations, can help clinicians align anticoagulation decisions with realistic time-to-benefit horizons and improve the clarity of documentation.

However, in frailty, trajectories are more protracted, with disability accumulating months to years before becoming relatively more disabled in the final year and especially dependent during the last month [32]. Many patients with multimorbidity may have similarly short prognostic horizons, yet often fall outside cancer-specific evidence and pathways. In these heterogeneous populations, fragmented ownership across transitions of care can further perpetuate continuation by default as responsibility for reassessing indication, prognosis, and reversal intent is not well defined. This variation further complicates antithrombotics decisions as time-to-benefit profile for stroke prevention in frail older adults may remain meaningful for longer than in rapidly progressive malignancy.

## 4 What is the Real-world Incidence of Thrombosis at the End-of-life?

Consecutive patients underwent bilateral femoral ultrasonography on admission and weekly for up to 2 weeks in the core Hospice Inpatient DVT Detection (HIDDen) study of people with advanced cancer admitted to specialist palliative-care units (SPCUs) in the United Kingdom [33]. It may be relevant to note that the criteria for admission to an SPCU in the United Kingdom is usually for complex symptom control or for those deemed to be in the last short weeks of life. Among 273 evaluable scans, 34% (92/273) of patients had femoral DVT at baseline. Only 4 additional patients without DVT at baseline developed a new femoral DVT over 21 days. Prior VTE, being bedbound in the previous 12 weeks, and lower-limb edema were independent predictors of DVT. Interestingly, the presence of DVT was not associated with survival, and neither thromboprophylaxis use nor serum albumin concentration showed associations with DVT status. The authors concluded that asymptomatic iliofemoral thrombosis is common on admission but conferred little symptom burden or survival disadvantage, challenging the routine application of hospital-style prophylaxis to SPCU populations with poor performance status whose short-term risk of incident VTE appears low even without primary prophylaxis. It also suggested that VTE in advanced patients with cancer was most likely part of the agonal process and not the ultimate cause of death [34]. The main implication of HIDDen is therefore a marked divergence between a high incidence of largely asymptomatic proximal DVT detected at hospice and SPCU admission and a very low short-term incidence of new proximal DVT during the subsequent hospice stay. This suggests that for many patients, the most clinically meaningful short-horizon outcomes may relate more to symptom burden and bleeding risk than to prevention of new thrombotic events within days to weeks.

A HIDDen exploratory substudy targeted SPCU in patients with advanced nonmalignant disease [35]. Using the same ultrasound protocol, 36% (8/22) of evaluable participants had a femoral DVT on admission. As in the cancer cohort, lower-limb edema or pain, breathlessness, and chest pain were high regardless of DVT status, underscoring that, in SPCU settings, symptoms are often multifactorial and that the mere presence of a proximal DVT does not reliably map onto symptom attribution or survival trajectories. A similar study examining thrombosis events in patients with cancers under SPCUs found the overall incidence of thrombosis was 22.2%, but with only 6.2% occurring after initiating SPCU care [36]. The authors suggest that blanket thromboprophylaxis may not be warranted as only a few developed symptomatic events. The data suggest that a sizable minority of SPCU admissions have silent proximal thrombosis acquired before admission but that the short-term risk of new events after admission is low even without systematic treatment escalation. In advanced disease, prevalent thrombosis appears to be a marker of the systemic inflammatory state and tumor biology, rather than a proximate cause of death, and attempts to transpose hospital thromboprophylaxis protocols to SPCU or hospice care may overestimate short-horizon benefits while underestimating treatment burdens and bleeding risks. HIDDen’s findings have already prompted prospective work to test whether the risk profile of palliative inpatients admitted to hospital (rather than hospice or SPCU) differs meaningfully from hospice cohorts. The HIDDen2 protocol aimed to determine whether hospital-based palliative admissions warrant a different approach to screening or prophylaxis because their short-term thrombotic risk could be higher than that seen after hospice admission [37].

In addition, population-level home palliative-care data complement the SPCU picture by following what happens when anticoagulation is continued or withdrawn over the subsequent months of community-based care. In a province-wide Ontario cohort of 98,089 older adults initiating home palliative care, 15.5% were taking an anticoagulant at the index palliative visit [38]. Among users, physician-driven discontinuation was common and, after adjustment, it was associated with similar rates of thrombotic events (adjusted hazard ratio, 1.06; 95% CI, 0.81-1.39), lower bleeding (adjusted hazard ratio, 0.75; 95% CI, 0.62-0.90), and a higher likelihood of dying at home (adjusted OR, 1.22; 95% CI, 1.09-1.36) compared with continuation. The findings suggest that withdrawing anticoagulation does not appear to precipitate excess thrombosis, may reduce harms, and help align place of death with preferences.

For practice, these data support an argument of not instituting routine pharmacologic thromboprophylaxis for SPCU and hospice in patients with poor performance status and limited prognosis while remaining vigilant for symptomatic VTE that meaningfully worsens breathlessness or limb pain where time-limited therapeutic anticoagulation may still be of symptomatic benefit. They also justify structured anticoagulant review at the time of SPCU or hospice admission and at the first home palliative-care visit, making deprescribing a proactive consideration when prognosis is short, symptom benefit is unlikely, and bleeding or treatment burden is salient.

## 5 The Risk of Bleeding While on Anticoagulation

A prospective estimate of bleeding among palliative inpatients comes from the multicentre French Risque Hemmoragique En SOins palliatifs (RHESO) study, which followed up 1199 people admitted to 22 palliative-care units for advanced cancer or nonmalignant life-limiting illness [39]. Over 3 months, the competing-risk cumulative incidence of clinically relevant bleeding was 9.8%, with 23 patients experiencing fatal bleeding. Independent risk factors for bleeding were active cancer, recent bleeding before admission, pharmacologic thromboprophylaxis, and concomitant antiplatelet therapy. By contrast, incident symptomatic DVT was rare (0.5%), underscoring a mismatch between the frequency of bleeding and the short-horizon thrombotic benefit in this setting. The findings of the RHESO study supports daily, goal-concordant review for palliative care in patients in whom prognosis is short and performance status is poor.

Observational cohorts also reinforce the central message that bleeding is common while on antithrombotics in the last months of life and that deprescribing late does not appear to precipitate a surge in thrombotic events (Table 3) [[38], [39], [40], [41], [42]]. In a Swedish single-center retrospective cohort encompassing the last year of life for 1501 palliative-care patients, 897 received antithrombotics, of whom, 16% (144/897) experienced at least 1 bleeding event while on therapy [40]. In adjusted models, men with prostate cancer had higher bleeding risk than those with other cancers (adjusted relative risk, 1.9; 95% CI, 1.1-3.2), whereas no significant differences emerged by drug class or indication. A large majority of patients (56%) remained on treatment until the last 3 days of life, and only 0.2% experienced stroke after deprescribing. Similarly, a Canadian palliative residence cohort reported that 40% of residents were on anticoagulation at or within 30 days of admission [41]. Of which, 78% of users discontinued during the stay. The cumulative probability of bleeding during the palliative stay was 7.3% overall, with higher rates reported among those who continued anticoagulation (10.8%) vs those who stopped (7.6%). The authors estimated a 44% increased bleeding risk with continued therapy against a 33% relative reduction in suspected VTE. Similar studies have also found significant bleeding risk without additional benefits of preventing VTE symptoms at the end-of-life [42]. The evidence suggests that over the short admission horizon, clinically relevant bleeding occurs in ∼1 in 10 patients, and fatal hemorrhage, although less common, does occur. Symptomatic VTE remains uncommon, and any symptoms attributed to VTE can be managed using end-of-life medicines. Clinicians should therefore consider discontinuation of anticoagulation at the end-of-life stage, particularly where the indication is long-term primary prevention and no clear short-horizon symptomatic benefit is expected.

**Table 3:** Observational data on bleeding risk associated with antithrombotics at end-of-life.

Study	Sample size	Setting	Time horizon	Bleeding outcomes	Thrombosis outcomes
Tardy et al. [39]	1199	Inpatient palliative-care units	3-mo follow-up	Clinically relevant bleeding, 9.8%	Symptomatic DVT 0.5%
Frisk et al. [40]	1501 (897 on antithrombotics)	Palliative-care service (mixed setting)	Last year of life	≥1 bleeding episode, 16%	Stroke after deprescribing, 0.2%
Polesello et al. [41]	453	Hospice or palliative residence	During residence stay	Overall bleeding, 7.3% Continue AC, 10.8%, vs stop AC, 7.6%	Overall suspected VTE, 5.7% continue AC, 4.6%, vs stop AC, 6.7%
Noble et al. [42]	214 patients with CAT	Specialist palliative-care service	Final weeks of life	CRNM bleeding, 7%	No symptomatic VTE events recorded
Chin-Yee et al. [38]	8687	Home or community palliative care	After first home palliative visit	Stopping AC associated with lower bleeding (aHR, 0.75)	Similar thrombosis risk (aHR, 1.06)

AC, anticoagulation; aHR, adjusted hazard ratio; CAT, cancer-associated thrombosis; CRNM, clinically relevant nonmajor (bleeding); DVT, deep vein thrombosis; VTE, venous thromboembolism.

## 6 How can we Safely Deprescribe Anticoagulation?

The evidence in the literature suggests the following:

1. Routine primary pharmacologic prophylaxis for SPCU or hospice patients with very limited prognosis is hard to justify on present evidence. If considered at all, it should be targeted to individuals whose short-term incident risk and goals-of-care make benefit plausible despite bleeding risk.
2. Whenever secondary anticoagulation is continued, clinicians should anticipate bleeding and actively deprescribe antiplatelets without a compelling indication.
3. Deprescribing should be a proactive, structured option as trajectories shorten, with clear documentation of a discussion with the patient about the accepted risks and reversal intent.

**Figure 1:**
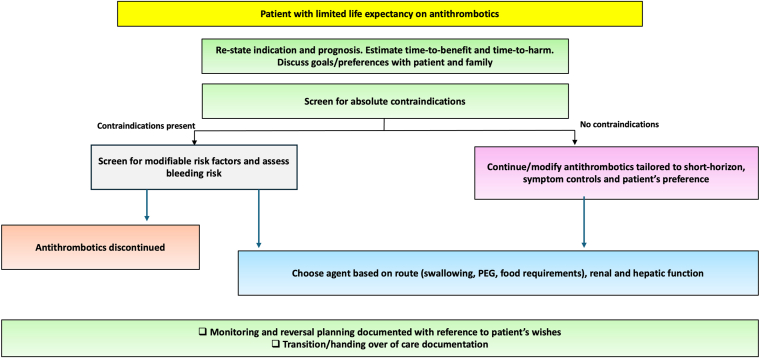
Framework for decision making on antithrombotic therapy at the end-of-life. In those with limited life expectancy, the decision to initiate or continue antithrombotic therapy should start with restating the indication and prognosis, estimating time-to-benefit vs time-to-harm profiles, clarifying patient and family goals, and screening for bleeding risk and contraindications. Depending on these considerations, therapy may be continued, modified, or discontinued. The framework should be interpreted according to the underlying indication as the expected benefit of antithrombotic therapy varies substantially between settings such as atrial fibrillation and venous thromboembolism.

A coherent deprescribing stance begins with acknowledging the mismatch between time-to-benefit and time-to-harm profiles in serious illness (Figure 1). For example, benefits from anticoagulation for AF accrue slowly, whereas bleeding risk, monitoring requirements, and drug interactions manifest early. Accordingly, end-of-life anticoagulation decisions should be framed as a shared decision-making process with the patient and, where appropriate, family, explicitly exploring goals, fears, and acceptable trade-offs. Trials of structured anticoagulation decision aids show that such shared decision making improves patient involvement, risk comprehension, and anxiety [43]. One approach suggested is that end-of-life decisions should be anchored in prognosis estimation, an explicit discussion of goals and preferences with the patient, and an indication-specific appraisal of time horizons [44]. It should then be checked against bleeding risks and practicalities before choosing to continue, time-limit, de-escalate, or stop therapy.

## 7 Can Deprescribing Tools Help?

Alongside these indication-level judgments, 2 sets of deprescribing tools provide guidance for teams who must revisit long-standing medicines in patients with limited life expectancy. In oncology palliative care, the OncPal Deprescribing Guideline offers a validated set of triggers to discontinue preventive drugs for which benefits are unlikely to materialize within the patient’s horizon. Antithrombotic agents, such as aspirin, are treated in context (continue for short-horizon symptomatic benefit and deprescribe for long-horizon primary prevention), with strong concordance against expert panels in the original validation [45]. However, it is lacking strong recommendation relevant to anticoagulation specifically [46]. In advanced frailty, Screening Tool of Older Persons Prescriptions in Frail adults with limited life expectancy (STOPPFrail) and its version 2 update supply explicit criteria and a process for medication review in adults with limited life expectancy [47]. Randomized and implementation studies show that applying STOPPFrail reduces medication count without worsening clinical outcomes and quality of life [48], supporting systematic reconsideration of antithrombotics alongside other long-term preventives. However, these tools were not specifically developed or validated for anticoagulation decisions across all indications, which reinforces the need for individualized judgment rather than algorithmic application.

Despite their limitations, such tools should be constantly used in practice in this setting. NICE guidelines advise daily reassessment of VTE prophylaxis for hospitalized palliative-care patients [4], but in reality, the commonest times to reassess medication are on admission and prior to discharge when the drugs to take home are being prescribed. The deprescribing frameworks mentioned recommend revisiting medication choices whenever performance status, bleeding/thrombotic risk, or goals-of-care change. For someone with months-long prognosis and reasonable function, continuing anticoagulation to prevent stroke or recurrent VTE may be appropriate, but as functional decline accelerates or prognosis shortens into weeks, teams should explicitly reevaluate and often simplify or stop therapy in line with updated goals.

Shared decision making is crucial in this context. Clinicians should explain the indication and clarify patient’s and family’s values or preferences [44]. The Anticoagulation Choice trial demonstrated higher shared decision-making quality and better risk comprehension when clinicians used a structured aid during anticoagulation conversations [43]. Subsequent trials confirm reductions in decisional conflict with shared decision-making tools in anticoagulation clinics. For end-of-life anticoagulation specifically, the Towards Cancer Patient Empowerment of Optimal Use of Antithrombotic Therapy at End of Life (SERENITY) program is developing and testing an information-driven shared decision support tool to guide appropriate (dis)continuation of antithrombotic therapy in people with cancer at the end-of-life [49]. It is explicitly targeting prescribing inertia and communication pitfalls that keep patients on therapy of uncertain value.

## 8 What if Anticoagulation is Still Pursued?

### 8.1 Ensure no contraindications

**Figure 2:**
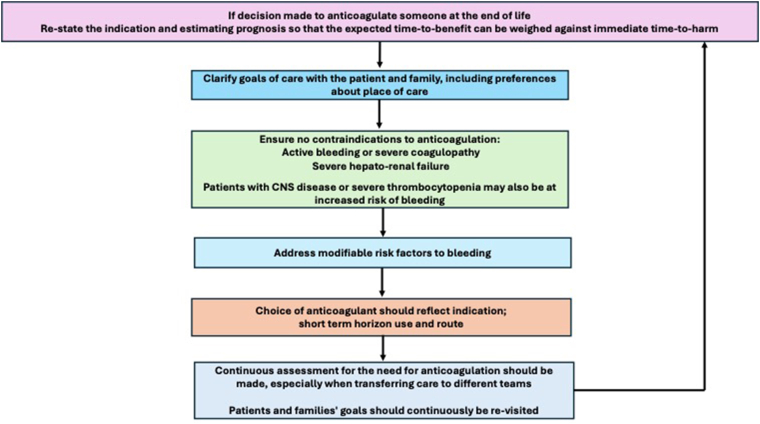
Suggested approach when a decision is made to continue anticoagulation at the end-of-life. Several factors should be considered if the decision is taken to continue anticoagulation at the end-of-life. Ongoing reassessment is essential, particularly during clinical deterioration or transfer between care settings. It is crucial to ensure that anticoagulation remains aligned with prognosis, feasibility, and patient preferences throughout each step.

If a decision is made to anticoagulate an individual approaching the end-of-life, the first step is to ensure that no absolute contraindication is present (Figure 2). General contraindications include active, uncontrolled bleeding, severe coagulopathy, and severe hepatorenal failure. In a study of older patients with AF, those ineligible for anticoagulation due to contraindication were more likely to have dementia, heart failure, or a higher CHADS2 score [50]. Patients with cancer are more likely to bleed on anticoagulation, and the risk is increased in the presence of central nervous system tumors and metastases [[51], [52], [53], [54]]. Other potential risk factors include chronic kidney disease and thrombocytopenia of < 50 × 10^9^/L. Progressive frailty also brings increasing risk of falls and trauma; which clinicians often cite as a reason to avoid or stop anticoagulation [55]. However, studies in older adults suggest that high fall risk alone does not significantly increase major bleeding risk [56,57]. Yet, in patients with recurrent injurious falls, cognitive impairment or prior intracranial hemorrhage and a short prognosis, the possibility of traumatic intracranial bleeding from the falls may need to be considered as a significant factor toward deprescribing in the shared decision-making process.

Once absolute contraindications are excluded, potentially modifiable bleeding risks should be addressed before or alongside prescribing. The priorities are to stop nonessential antiplatelet agents and nonsteroidal anti-inflammatory drug and offer proton pump inhibitor protection where indicated.

### 8.2 Appropriate anticoagulant to be chosen

With respect to choice of agent, the same therapeutic options exist as in longer-horizon care, but end-of-life practice adds distinctive practical constraints that often complicate decision making. Wherever possible, warfarin should be avoided due to its drug–drug interaction, high burden of monitoring, and unpredictable bleeding associated with dietary fluctuations. While there are certain conditions where only warfarin is indicated for anticoagulation (such as stroke prophylaxis in patients with mechanical heart valves), in general, warfarin should be avoided in patients with advanced cancer. Despite clinical guidelines recommending low-molecular-weight (LMWH) or direct oral anticoagulants (DOACs) over vitamin K antagonists for the treatment of cancer-associated VTE, warfarin prescribing remains significant, particularly in insurance-based health care settings [58]. There is a plethora of data, which shows warfarin to have a higher rate of recurrent VTE when compared with LMWH in the cancer cohort, albeit with no statistical difference in major bleeding [59]. As intake wanes and the malignant hypercatabolic state progresses, warfarin control becomes more labile owing to variable vitamin K consumption and reduced hepatic synthesis of clotting factors. Real-world data in patient with advanced cancer reports the international normalized ratio (INR) to be difficult to control and is associated with high bleeding rates. Tighter anticoagulation control requires frequent monitoring (every 2 days) and impact on patients’ quality of life [60,61].

Dosing schedule and route of administration matter as fatigue, nausea, and dysphagia become common. Some patients, for example, may prefer a once-daily subcutaneous LMWH because it bypasses the gastrointestinal tract and avoids the nausea or pill burden experienced with oral agents, while others may prefer an oral regimen while swallowing is intact. In the oral class, DOACs differ in ways that are particularly relevant when intake is irregular. Rivaroxaban at therapeutic doses is better absorbed with food [62], a requirement that can be difficult to meet when appetite is poor or feeds are interrupted. By contrast, apixaban can be given without regard to meals [63]. Where enteral tubes are in place, apixaban and rivaroxaban may be crushed and administered via nasogastric or gastric tubes with acceptable bioavailability if the tube terminates in the stomach [64], but the benefits of continuing a medication just because it can be put down a tube need to be considered. Dabigatran, because it is a prodrug formulated for acid-dependent absorption, should not be opened or crushed and is not recommended for administration through feeding tubes [64]. If rivaroxaban is given through a feeding tube, the 15- to 20-mg doses should be coadministered with nutrition, and tubes that bypass the stomach are discouraged because of reduced absorption [64]. DOACs and LMWH exposure increases when renal function declines [65], raising bleeding risk. It may be prudent to undertake close, iterative reassessment of dose, route, and even the need to continue therapy at all.

### 8.3 Importance of patient preference

It is good clinical practice to regularly review the medications of patients with advanced cancer or progressive chronic disease in order to decrease tablet burden and reduce the risk of drug–drug interactions and adverse events from drug accumulation as organ systems shut down. Consideration of patient preferences is of utmost importance with respect to anticoagulation since their views on whether to continue or cease anticoagulation will be strongly influenced by their previous experiences of VTE and anticoagulation. For example, a patient who experienced a symptomatic VTE event will be more inclined to continue anticoagulation than a patient anticoagulated for an incidental thrombus. Similarly, a patient with a history of major or clinically relevant nonmajor bleeding may favor discontinuation more than someone with no experience of hemorrhage. A management plan outlining patient preference in the event of bleeding symptoms should be discussed and agreed at the time of prescribing anticoagulants or reviewing medication for an individual approaching the end-of-life. Considerations include the drug half-life, with warfarin being over a number of days and DOACs and LMWH typically much shorter, although dependent on renal function. Reversal options, such as community-administered vitamin K for warfarin, should be considered. Acute bleeding would require hospitalization for acute reversal [66]. A patient nearing the end-of-life should have the opportunity to discuss their preferences regarding admission for bleed management and what symptoms should trigger a call for help. Anchoring therapy to a clear reversal intent avoids distressing uncertainty should bleeding occur. Counter to these thrombotic events should also be considered and a plan to stop anticoagulation including a plan regarding treatment or not if a thrombosis occurs.

Finally, patients who wish to discontinue anticoagulation should be supported to do so using a structured deprescribing approach as explored earlier. Deprescribing remains a planned, clinically supervised process of withdrawing a medication that is no longer indicated or is causing disproportionate harm. There is a need to clarify the indication and time-to-benefit profiles, identify and mitigate withdrawal or rebound risks, document the rationale, and communicate the plan to all teams. In the context of anticoagulation, that typically includes recording whether thrombotic events will be treated if they occur, whether hospital transfer for reversal will be pursued in the event of a bleed, and what symptoms should trigger a call for help.

### 8.4 During transitions

Ongoing prescribing and monitoring responsibilities should be explicit at every transition. In hospital, the responsible team should confirm indication, prognosis, and reversal intent before discharge. In the community, general practitioners or primary physicians typically assume prescribing with hospice or community nursing support for LMWH administration and symptom monitoring. Anticoagulation clinics or cardiology provide INR oversight only if that service remains feasible and aligned with goals. Where care shifts to hospice or a nursing home, handover notes should state the indication, prognosis band, bleed-risk amplifiers, route or feasibility plan (eg, nasogastric tube–compatible agent), and the agreed plan for monitoring and reversal.

Hospice and community teams need shared checklists that translate values and risk framing into safe practice during transitions of care. The National Hospice and Palliatice Care Organization (NHPCO) Hospice Medication Deprescribing Toolkit, adopted in several US hospice programs, documents the conversation and requirements specifically for antiplatelets and anticoagulants [67]. It confirms indication and prognosis, reconcile goals, reviews bleeding risks, and verify feasibility (eg, crushability, enteral access, and monitoring), and record the team’s intent regarding reversal agents. It also sets expectations about discontinuing warfarin when INR monitoring is no longer acceptable or feasible and about de-escalating when ongoing use is outside goals-of-care. These implementation prompts are particularly important at hospital–hospice or hospital–nursing-home discharge, when prescribing inertia and fragmented ownership often perpetuate antithrombotic therapy by default.

### 8.5 Summary

When put together, steps around antithrombotic use in this setting may be summarized as follows:

1. Begin by restating the indication and estimating prognosis so that the expected time-to-benefit profile can be weighed against immediate time-to-harm profile.
2. Clarify goals-of-care with the patient and family, including preferences about place of care.
3. Screen for absolute contraindications and risk factors for bleeding and address what is modifiable.
4. Decide between continuing as is for a clearly short-horizon benefit, a time-limited course with a defined reassessment point or discontinue entirely. The decision should be anchored in what matters most to the patient.
5. Document the plan with explicit statements about reversal intent, whether hospital transfer would be pursued for a bleed or thrombosis, how symptoms will be managed if events occur, and who is responsible for monitoring.
6. Review at each clinical change, with a low threshold to simplify further or to deprescribe completely as function declines, swallowing fails, monitoring becomes impractical, or goals shift toward comfort alone.

## 9 Conclusion

Antithrombotics at the end-of-life should be governed by a proactive appraisal of prognosis, time-to-benefit vs time-to-harm profiles, feasibility, and what matters most to the individual and family. However, the evidence behind most of these recommendations remains predominantly observational. The most relevant data indicate that incident thrombotic events are uncommon during short hospice admissions, while bleeding is frequent and distressing. Deprescribing in community palliative care is often safe and may better align care with patient preferences. If anticoagulation is continued for clear near-term benefit, clinicians should mitigate modifiable bleeding risks and set an explicit reversal plan consistent with goals. The recommendations in this review should be interpreted in the context of the specific indication, as time-to-benefit varies substantially across antithrombotic therapies. Accordingly, the balance between potential benefit, bleeding risk, and treatment burden will also differ significantly. Key unknowns include which patients gain meaningful symptom benefit within short prognostic horizons and how best to individualize decisions using end-of-life–appropriate risk and decision-making tools. Decisions and transitions should therefore be documented with clarity around indication, horizon, monitoring responsibilities, and, importantly, revisited as the clinical picture evolves. Current knowledge supports an approach that privileges shared decision making and judicious deprescribing to maximize benefit and minimize harm in the time that remains.
